# Dexmedetomidine alleviates cognitive impairment by promoting hippocampal neurogenesis via BDNF/TrkB/CREB signaling pathway in hypoxic–ischemic neonatal rats

**DOI:** 10.1111/cns.14486

**Published:** 2023-10-13

**Authors:** Xiaohui Chen, Andi Chen, Jianjie Wei, Yongxin Huang, Jianhui Deng, Pinzhong Chen, Yanlin Yan, Mingxue Lin, Lifei Chen, Jiuyun Zhang, Zhibin Huang, Xiaoqian Zeng, Cansheng Gong, Xiaochun Zheng

**Affiliations:** ^1^ Department of Anesthesiology Shengli Clinical Medical College of Fujian Medical University, Fujian Provincial Hospital Fuzhou China; ^2^ Fujian Provincial Key Laboratory of Emergency Medicine Fuzhou China; ^3^ Fujian Emergency Medical Center, Fujian Provincial Key Laboratory of Critical Care Medicine Fujian Provincial Co‐Constructed Laboratory of “Belt and Road” Fuzhou China

**Keywords:** astrocyte, BDNF, cognitive impairment, hippocampal neurogenesis, hypoxic–ischemic brain damage

## Abstract

**Aims:**

Dexmedetomidine (DEX) has been reported to alleviate hypoxic–ischemic brain damage (HIBD) in neonates. This study aimed to investigate whether DEX improves cognitive impairment by promoting hippocampal neurogenesis via the BDNF/TrkB/CREB signaling pathway in neonatal rats with HIBD.

**Methods:**

HIBD was induced in postnatal day 7 rats using the Rice‐Vannucci method, and DEX (25 μg/kg) was administered intraperitoneally immediately after the HIBD induction. The BDNF/TrkB/CREB pathway was regulated by administering the TrkB receptor antagonist ANA‐12 through intraperitoneal injection or by delivering adeno‐associated virus (AAV)‐shRNA‐BDNF via intrahippocampal injection. Western blot was performed to measure the levels of BDNF, TrkB, and CREB. Immunofluorescence staining was utilized to identify the polarization of astrocytes and evaluate the levels of neurogenesis in the dentate gyrus of the hippocampus. Nissl and TTC staining were performed to evaluate the extent of neuronal damage. The MWM test was conducted to evaluate spatial learning and memory ability.

**Results:**

The levels of BDNF and neurogenesis exhibited a notable decrease in the hippocampus of neonatal rats after HIBD, as determined by RNA‐sequencing technology. Our results demonstrated that treatment with DEX effectively increased the protein expression of BDNF and the phosphorylation of TrkB and CREB, promoting neurogenesis in the dentate gyrus of the hippocampus in neonatal rats with HIBD. Specifically, DEX treatment significantly augmented the expression of BDNF in hippocampal astrocytes, while decreasing the proportion of detrimental A1 astrocytes and increasing the proportion of beneficial A2 astrocytes in neonatal rats with HIBD. Furthermore, inhibiting the BDNF/TrkB/CREB pathway using either ANA‐12 or AAV‐shRNA‐BDNF significantly counteracted the advantageous outcomes of DEX on hippocampal neurogenesis, neuronal survival, and cognitive improvement.

**Conclusions:**

DEX promoted neurogenesis in the hippocampus by activating the BDNF/TrkB/CREB pathway through the induction of polarization of A1 astrocytes toward A2 astrocytes, subsequently mitigating neuronal damage and cognitive impairment in neonates with HIBD.

## Graphical abstract

DEX promoted hippocampal neurogenesis by promoting the polarization of A1 astrocytes to A2 astrocytes and then activating BDNF/TrkB/CREB signaling pathway, thus alleviating brain injury and long‐term cognitive impairment in neonatal HIBD rats. DEX, dexmedetomidine; HIBD, hypoxic–ischemic brain damage; By Figdraw.

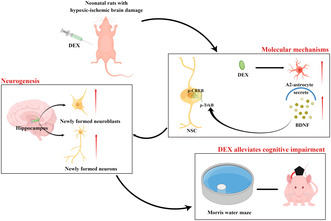

## 1 INTRODUCTION

Hypoxic–ischemic brain damage (HIBD) is a leading cause of neurodevelopmental disorder in human neonates, occurring in 2–3 per 1000 newborn children.^1^ The majority of neonates who survive HIBD experience permanent neurological deficits, including audiovisual impairment, cerebral palsy, and cognitive impairment.^2^, ^3^ Nevertheless, the mechanisms underlying HIBD‐induced cognitive impairment remain incompletely elucidated, and effective preventive interventions have yet to be identified.

The hippocampus is a crucial brain structure for cognition, particularly in learning and memory processes.^4^ The hippocampus is known to be more susceptible to cerebral hypoxia and ischemia than other brain regions, leading to a heightened risk of neuronal damage in neonates,^5^ therefore, we chose to focus our study on the hippocampus. Previous studies, including our own research,^6^, ^7^, ^8^ have demonstrated that neonatal hypoxia‐ischemia results in substantial neuronal damage and neurological deficits through a multifaceted interplay of pathways, involving oxidative stress, excitotoxicity, and neuroinflammatory reactions. Neurogenesis in the hippocampus plays a critical role in neuronal plasticity and cognitive function. It is widely recognized that new neurons continue to be generated in specific regions of the brain, such as the hippocampal dentate gyrus subgranular zone and subventricular zone throughout the lifespan.^9^ Multiple animal studies have provided evidence that newly generated neurons originating from the dentate gyrus have the ability to migrate toward the granule cell layer and contribute to neural repair and cognitive recovery.^10^, ^11^ However, the potential of spontaneous endogenous neurogenesis for repairing and regenerating the brain is limited and insufficient to compensate for neuron loss after HIBD.^12^ Therefore, enhancing hippocampal neurogenesis may serve as a promising treatment strategy for alleviating neurological deficits induced by neonatal HIBD. Brain‐derived neurotrophic factor (BDNF), a member of the neurotrophin family, is essential for the differentiation of neural stem cells (NSCs), as well as neuronal growth, maturation, and maintenance.^13^, ^14^ BDNF facilitates hippocampal neurogenesis by binding to the tyrosine kinase receptor B (TrkB). Subsequently, BDNF/TrkB signaling initiates the activation of the phosphorylated cAMP response element‐binding protein (p‐CREB), which plays a crucial role in stimulating the transcription of genes involved in hippocampal neurogenesis.^15^, ^16^, ^17^ Recent studies have shown that activated astrocytes play a significant role as a source of BDNF following brain injury.^18^ It is important to note that activated astrocytes can adopt two different phenotypes^19^: A1 astrocytes secrete neurotoxic factors that mediate the classical complement cascade, leading to the expansion of neuroinflammation. Conversely, A2 astrocytes upregulate the expression of various neurotrophic factor proteins, including BDNF, which exert neuroprotective effects.^19^, ^20^ Therefore, therapeutic interventions that promote the conversion of astrocytes from a neurotoxic A1 phenotype to a neuroprotective A2 phenotype, thereby increasing BDNF secretion in the hippocampus following brain injury, could confer neuroprotection through activation of the BDNF/TrkB/CREB pathway.

Dexmedetomidine (DEX) is a highly selective α‐2 adrenergic receptor agonist with analgesic, sedative, and anxiolytic effects. It is extensively utilized in intensive care and perioperative settings.^21^ Our previous studies have demonstrated that pretreatment or posttreatment with DEX could effectively alleviate neuronal damage and neurological deficits in neonates following HIBD.^7^ Moreover, DEX has been shown to have a beneficial impact on neurogenesis after various types of brain injury, such as ketamine and midazolam‐induced neurotoxicit.^22^, ^23^ However, there is limited understanding regarding the underlying intrinsic mechanisms of DEX‐mediated hippocampal neurogenesis and its impact on cognitive impairment induced by HIBD.

Accordingly, the present study aimed to investigate the effect of DEX treatment on hippocampal neurogenesis and astrocyte polarization in neonates following HIBD, as well as its regulatory mechanism on the BDNF/TrkB/CREB signaling pathway. Additionally, we conducted further research to examine the involvement of the BDNF/TrkB/CREB pathway in DEX‐mediated neurogenesis and neuroprotection. Specifically, we administered the TrkB receptor antagonist ANA‐12 through intraperitoneal injection and delivered an adeno‐associated virus (AAV) carrying shRNA targeting BDNF via intrahippocampal injection.

## 2 MATERIALS AND METHODS

### 2.1 Animals and ethics statement

This research has been approved by the Animal Care and Use Committee at Fujian Medical University (Fuzhou, China) (No: IACUCFJMU 2022–0463). Lab animals were handled according to the Guide for the Care and Use of Laboratory Animals. During the experiment, we housed the neonatal rats in cages with their littermates and nursing rats under controlled temperature and light conditions (12 h of light/12 h of darkness) and provided them free access to food and water.

### 2.2 Establishment of the neonatal HIBD model

We used the classical Rice–Vannucci modeling approach to construct the neonatal HIBD model as described previously.^24^ We first ligated the left common carotid artery on postnatal day 7 Sprague–Dawley (SD) rats under 3% isoflurane anesthesia. Next, we placed the neonatal rats into a hypoxic chamber filled with 8% O_2_ for 2 h at 37°C after 1 h of recovery next to their dams. This approach caused HI insults to neonatal rats.

### 2.3 Experimental protocol

First, we conducted high‐throughput RNA sequencing to explore the genes and molecular pathways involved in the potential neuromorphopathological mechanisms associated with HIBD. Neonatal rats were divided into two groups: (1) the sham group, in which the rats were anesthetized and their left common carotid artery was exposed without inducing hypoxia or ligation, and (2) the HIBD model group, referred to as the HI group. At 2 days post‐HI insults, we collected ipsilateral injury hippocampal tissues for high‐throughput RNA sequencing. Subsequently, we conducted western blot analysis to assess whether the protein expression changes aligned with the alterations in RNA expression.

Second, we investigated whether DEX provides neuroprotection in neonatal rats with HIBD by involving neurogenesis and the BDNF/TrkB/CREB pathway. The neonatal rats were divided into three groups: (1) the sham group, (2) the HI group, and (3) the HI + DEX group: The HIBD model rats were treated with DEX (25 μg/kg, Sigma‐Aldrich) via intraperitoneally injection immediately after HI insults. This dosage and mode of administration were chosen based on the findings of our previous studies on the neuroprotective effects of DEX.^6^, ^24^ We measured the expression levels of BDNF/TrkB/CREB pathway‐related proteins, observed changes in astrocytes phenotype, and assessed hippocampal neurogenesis.

Furthermore, in order to elucidate the specific involvement of BDNF/TrkB/CREB pathway in DEX‐mediated neurogenesis and neuroprotection in neonatal HIBD rats, we administered the TrkB receptor antagonist ANA‐12 through intraperitoneal injection. The neonatal rats were divided into four groups: (1) the sham group, (2) the HI group, (3) the HI + DEX group, and (4) the HI + DEX + ANA‐12 group: The neonatal rats received an intraperitoneal injection of ANA‐12 (0.5 mg/kg, HY‐12497, MCE) 4 h prior to the HI insults. Subsequently, the modeling procedure was performed, followed immediately by an intraperitoneal injection of DEX. Additionally, we used adeno‐associated viruses (AAV) carrying GFAP promoter (AAV–GFAP) to selectively knock down the BDNF gene in astrocytes of the ipsilateral hippocampus in neonatal rats through stereotactic injection. The neonatal rats were divided into four groups: (1) the sham group, (2) the HI group, (3) the HI + DEX + AAV‐GFAP‐Control‐EGFP viruses (AAV‐shCON) group, and (4) the HI + DEX + AAV‐GFAP‐BDNF(shRNA)‐EGFP viruses (AAV‐shBDNF) group: A stereotaxic injection of either AAV‐shCON or AAV‐shBDNF virus into the ipsilateral hippocampus was performed on postnatal day 1 rat. The modeling was then conducted on postnatal day 7, followed by an immediate intraperitoneal injection of DEX. We assessed the expression levels of proteins related to the BDNF/TrkB/CREB pathway, as well as evaluated hippocampal neurogenesis, brain injury, and learning and memory function.

### 2.4 Stereotactic injection (intrahippocampal injection)

Neonatal rats were immobilized using stereotactic frames while under 3% isoflurane anesthesia. A virus solution (1 μL) was then slowly injected into the ipsilateral hippocampus at specific coordinates relative to Bregma (ML = +1.8 mm, AP = −2.3 mm, and DV = −2.0 mm). The injection was performed using a micro‐syringe pump at a rate of 0.1 μL/min. The needle was subsequently removed 10 min following the completion of the infusion. The AAV‐shBDNF and AAV‐shCON viruses were purchased from Hanbio Tech, and the core sequence of AAV‐shBDNF was CAGUCAUUUGCGCACAACU.^25^

### 2.5 Bromodeoxyuridine labeling

Endogenous cell proliferation was evaluated using 5‐bromo‐2‐deoxyuridine (BrdU), a mitosis indicator. Two injections of BrdU (B5002, Sigma‐Aldrich, USA) were given intraperitoneally twice daily (12 h apart) at 4–6 days after HI insults, and we quantified neurogenesis in the hippocampus according to a previous study,^26^ rats were sacrificed at 14 and 28 days after HI insults to count newly formed neuroblasts and neurons in the hippocampus, respectively.

### 2.6 RNA extraction, library construction, and sequencing

At 2 days after HI insults, we euthanized neonatal rats from the sham and HI groups through cervical dislocation. We quickly collected ipsilateral hippocampal tissues, then snap‐froze them in liquid nitrogen and stored them at −80°C. We extracted total RNA using a TRIzol reagent kit (Invitrogen). Then, we performed RNase‐free agarose gel electrophoresis using an Agilent 2100 Bioanalyzer (Agilent Technologies) to verify the quality of the RNA extracted. Enriched mRNA was then reverse‐transcribed into cDNA using DNA polymerase I, RNase H, dNTPs, and buffer. Next, to purify the cDNA fragments and perform end‐repair, we used the QiaQuick PCR extraction kit (Qiagen). We added the poly(A) and attached the fragments to the Illumina sequencing adapters. We then carried out agarose gel electrophoresis to select the PCR amplification products. Sequencing was performed using the NovaSeq6000 from Gene Denovo Biotechnology Co.

### 2.7 Western blot analysis

Western blot analysis was performed as described previously.^7^, ^24^ The hippocampal tissues were collected at 1, 2, 3, 7, and 14 days after HI insults and immediately placed on ice. The tissues were then homogenized with a protease and phosphatase‐supplemented lysis buffer. Next, by sodium dodecyl sulfate–polyacrylamide gel electrophoresis, equal amounts of proteins (40 μg/well) were separated and transferred to polyvinylidene fluoride membranes. Subsequently, the membranes were incubated overnight at 4°C with the following primary antibodies: anti‐BDNF (ab108319, Abcam), anti‐TrkB (phospho Y705) (ab229908, Abcam), anti‐TrkB (ab187041, Abcam), anti‐CREB (phospho S133) (ab32096, Abcam), anti‐CREB (ab32515, Abcam) and anti‐β‐actin (ab8227, Abcam). The following day, the membranes were incubated at room temperature for 2 h with the secondary antibodies (ab205718, Abcam). We visualized and photographed protein bands using enhanced chemiluminescence substrate kits (ab133406, Abcam) and a GE Amersham Imager 600 (AI600; GE Healthcare). Full unedited gel/blot from this study can be found in Data S1.

### 2.8 Immunofluorescence staining

We utilized the markers C3 and S100A10 to identify A1 and A2 phenotype astrocytes, respectively. To determine the abundance of A1 astrocytes (GFAP^+^/C3^+^) and A2 astrocytes (GFAP^+^/S100A10^+^), we randomly selected one slice from each rat and counted the cells expressing GFAP, GFAP^+^/C3^+^, and GFAP^+^/S100A10^+^ in the dentate gyrus. Subsequently, we calculated the ratio of A1 astrocytes to the total number of astrocytes, as well as the ratio of A2 astrocytes to the total number of astrocytes. For neurogenesis assessment, we used the markers bromodeoxyuridine (BrdU) and doublecortin (DCX) to identify newly formed neuroblasts, and BrdU and neuronal nuclear protein (NeuN) to identify newly formed neurons. Similarly, we randomly selected one slice from each rat and quantified the number of BrdU^+^/DCX^+^ and BrdU^+^/NeuN^+^ cells in the dentate gyrus.

The immunofluorescence analysis was conducted as previously described.^24^ The brain tissues were collected and fixed in paraformaldehyde overnight at 4°C. Subsequently, the brain tissues were sectioned into 3.0–3.5 μm‐thick coronal sections and embedded in paraffin. We performed deparaffinization and hydration on the selected sections. Subsequently, a 0.1 M borate buffer solution (pH 8.5) was used to neutralize the medium after treating the denatured DNA with 2 N HCL for 30 min at 37°C. After blocking with 5% fetal bovine serum for 3 min, the sections were washed with phosphate‐buffered saline. We then incubated the sections overnight with the following primary antibodies: anti‐BDNF (ab108319, Abcam), anti‐GFAP (ab279289, Abcam), anti‐C3 (ab182890, Abcam), anti‐S100A10 (PA5‐95505, Invitrogen), anti‐doublecortin (Cell Signaling Technology), anti‐NeuN (MAB377, Millipore), anti‐BrdU (ab1893, Abcam) and anti‐GFP (A‐11120, Invitrogen). The following day, the sections were incubated with the appropriate secondary antibodies at room temperature for 2 h. Positive cells were manually counted by a skilled laboratory technician using a fluorescence microscope (Olympus FV1000) and the cell counter function of ImageJ 1.4.

### 2.9 Morris water maze test

We conducted the Morris water maze (MWM) test at 28 days after the HI insults to evaluate the cognitive function of rats as described previously.^6^ The MWM test consisted of a total of 6 days of testing. This included the place navigation test conducted over the first 5 days, followed by a probe trial on the 6th day. All rats were regularly tested between 9:00 a.m. and 3:00 p.m., avoiding disruptions due to diurnal variations and the influence of light on the experimental outcomes. Rat movements were captured by a video camera above the pool, which was divided into four quadrants with a platform (12 cm in diameter) submerged in one. The rats were allowed to swim freely in the pool for 90 s before the formal test. During the first 5 days (place navigation test), we placed the rats facing the pool wall and allowed them to search for the escape platform for 90 s. In event that rats failed to find the platform within 90 s, they were directed to it and allowed to remain there for 30 s. We conducted four consecutive experiments each day for 5 days and measured the average escape latency to assess spatial learning and memory ability. On the 6th day (probe trial test), we removed the hidden platform and let the rats swim in the pool for 90 s. As part of our assessment of their spatial memory ability, we recorded the number of times they crossed the platforms and the amount of time they spent in the target quadrant.

### 2.10 Nissl staining

We utilized Nissl staining to evaluate neuronal loss and the morphology of neurons in the sub‐granular zone (SGZ) of the dentate gyrus in the hippocampus. Following the MWM test, all rats were sacrificed, and brain tissues were immediately collected. These brain tissues were then processed through paraffin embedding and section‐cutting techniques. Subsequently, the dewaxed and dehydrated sections were stained with Cresyl violet acetate for a duration of 5–10 min, followed by two 10 s washes with distilled water. Finally, the sections were dehydrated twice for 5 min with serial ethanol and xylene, and then mounted on glass slides. The number and morphology of neurons were observed and analyzed using a light microscope.

### 2.11 Triphenyl tetrazolium chloride staining

We evaluated the size of cerebral infarcts by Triphenyl tetrazolium chloride staining (TTC) staining after the MWM test. We collected the brain tissues of rats immediately after sacrificing them under deep anesthesia. Next, we froze the brain tissues at −20°C for 20 min and cut them into 2 mm coronal slices. We then immersed the brain sections in a 4% TTC solution (Sigma‐Aldrich) in the dark for 30 min. Finally, the brain sections were fixed with 4% formaldehyde and analyzed. The calculation for infarct volume is derived as follows: (volume of the normal hemisphere ‐ volume of non‐infarct region in the affected hemisphere) divided by the total volume of the hemisphere, multiplied by 100%.

### 2.12 Statistical analysis

The data were analyzed using SPSS 22.0 software (SPSS Inc.). The distribution of the data was assessed for normality using the Shapiro–Wilk test, while the homogeneity of variance was evaluated using Levene's test. When the data was normally distributed, it was presented as mean ± standard deviation. To compare two groups, the student's *t*‐test was employed. For comparisons among three or more experimental groups, a one‐way analysis of variance (ANOVA) was conducted, followed by Bonferroni's post hoc test if the data adhered to a normal distribution. However, if the data did not conform to a normal distribution, a nonparametric Kruskal–Wallis test was utilized. The escape latency in the MWM test was assessed using a repeated measure two‐way ANOVA with “day” as the within‐subject factor and “group” as the between‐subject factor. *p* < 0.05 as statistically significant.

## 3 RESULTS

### 3.1 DEX treatment increased the expression of BDNF in the hippocampus after HIBD

**FIGURE 1:**
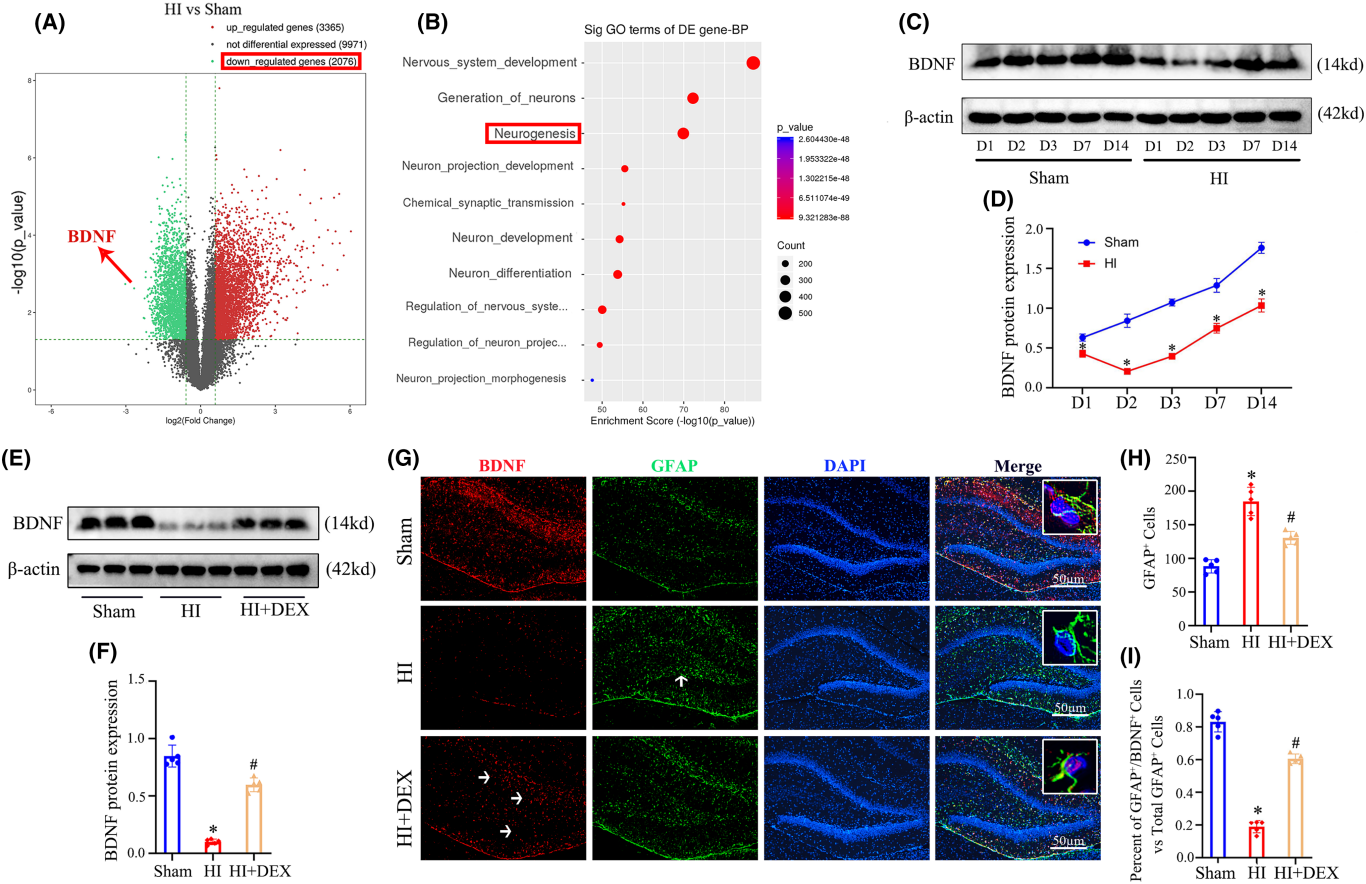
DEX treatment increased the expression of BDNF in the hippocampus of neonatal rats following HIBD. (A) Volcano plots display the DEGs as red dots (upregulated) and blue dots (downregulated). (B) The top 10 downregulated DEGs are categorized into classes based on GO enrichment terms for the biological process. (C, D) Western blot analysis was performed to examine the expression levels of BDNF at 1, 2, 3, 7, 14 days after HI. (E, F) Western blot analysis was performed to examine the expression levels of BDNF at 2 days after HI insults and DEX treatment. (G) Immunofluorescent staining was performed to examine the expression levels of BDNF in astrocyte of the dentate gyrus in the hippocampus. (H) Quantification for the number of GFAP^+^ cells in the dentate gyrus of the hippocampus. (I) Quantification for the ratio of BDNF^+^/GFAP^+^ cells to the total number of GFAP^+^ cells in the dentate gyrus of the hippocampus. DEGs, differentially expressed genes; DEX, dexmedetomidine; HI, hypoxic‐ischemia; HIBD, hypoxic‐–ischemic brain damage. Data were expressed as the mean ± SD (*n* = 5 per group); *p < 0.05 vs. the sham group; ^*#*^ *p* < 0.05 vs. the HI group.

We initially performed high‐throughput RNA sequencing to identify the differentially expressed genes (DEGs) in the hippocampus 2 days after HI insults. As shown in Figure 1A, we identified 3365 genes that were upregulated and 2076 genes that were downregulated in the HI group in comparison to the sham group. Notably, BDNF exhibited a significant decrease among these genes. Moreover, the analysis of gene ontology (GO) enrichment demonstrated that the DEGs were primarily associated with processes related to nervous system development, generation of neurons, and neurogenesis (Figure 1B), subsequently, we utilized western blot analysis to quantify the expression of BDNF protein in the hippocampus at various time points (1, 2, 3, 7, and 14 days) following HI insults (Figure 1C). The data showed a notable decrease in BDNF protein expression levels in the HI group compared to the sham group, with the most significant reduction observed 2 days after HI insults (Figure 1D). Consequently, we selected this time point to further investigate the impact of DEX treatment on BDNF expression and downstream signaling molecules in neonatal rats following HIBD.

As depicted in Figure 1E, we observed a significant increase in BDNF protein expression in the hippocampus of neonatal HIBD rats following DEX treatment (Figure 1F). In light of the fact that activated astrocytes play a crucial role as a source of BDNF post‐brain injury.^18^, ^27^ We then aimed to determine whether the increased protein levels of BDNF were associated with elevated BDNF expression in hippocampal astrocytes. The co‐localization of BDNF and astrocytes was evident in Figure 1G. In addition, HI insults induced substantial activation of astrocytes in the dentate gyrus of the hippocampus, which was effectively mitigated by DEX treatment (Figure 1H). Furthermore, the HI insults led to a significant decrease in BDNF‐positive astrocytes compared to the sham group, whereas DEX treatment resulted in a significant increase in BDNF‐positive astrocytes (Figure 1I).

### 3.2 DEX treatment promoted polarization of astrocytes from the A1 to A2 phenotype in the hippocampus after HIBD

**FIGURE 2:**
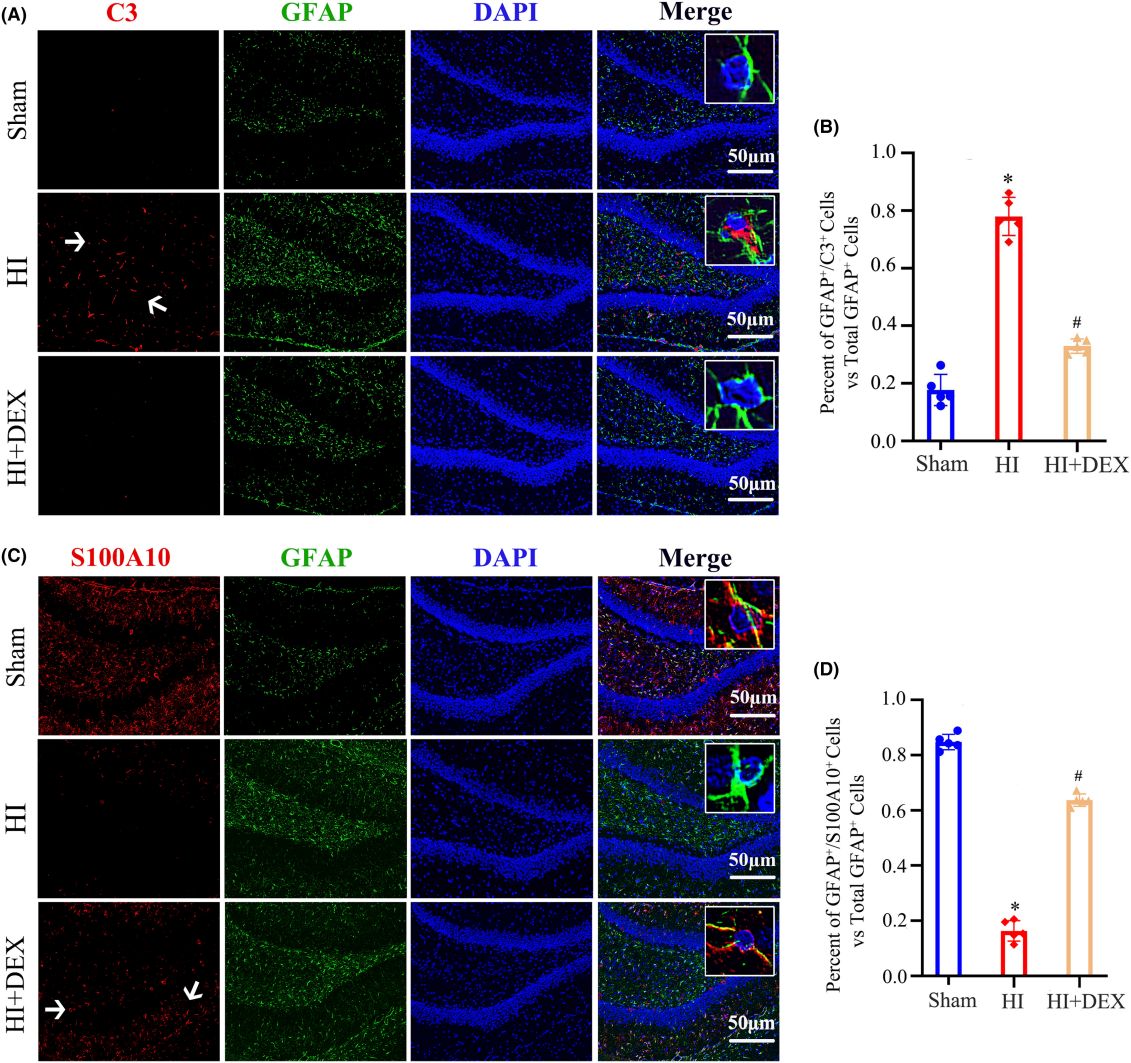
DEX treatment promoted polarization of astrocytes from the A1 to A2 phenotype in the hippocampus of neonatal after HIBD. (A) Dual immunofluorescence staining was performed to examine the number of A1 astrocytes labeling C3 and GFAP. (B) Quantification for the ratio of C3^+^/GFAP^+^ cells to the total number of GFAP^+^ cells in the dentate gyrus of the hippocampus. (C) Dual immunofluorescence staining was performed to examine the number of A2 astrocytes labeling S100A10 and GFAP. (D) Quantification for the ratio of S100A10^+^/GFAP^+^ cells to the total number of GFAP^+^ cells in the dentate gyrus of the hippocampus. DEX, dexmedetomidine; HI, hypoxic–ischemia; HIBD, hypoxic–ischemic brain damage. Data were expressed as the mean ± SD (*n* = 5 per group); ^*#*^ *p* < 0.05 vs. the HI group.

Multiple studies^28^, ^29^ have reported the neuroprotective effects of A2 astrocytes, which contribute to tissue repair through the secretion of various neurotrophic factors, including BDNF. BDNF plays a crucial role in promoting cell survival, nerve regeneration, synaptic plasticity, and memory formation in the hippocampus. We utilized immunofluorescence to determine the phenotype of activated astrocytes in the dentate gyrus of the hippocampus in neonatal rats following HI insults and DEX treatment. Our findings indicated that treatment with DEX leads to a decrease in the proportion of detrimental A1 astrocytes (Figure 2A,B) and an increase in the proportion of beneficial A2 astrocytes (Figure 2C,D) within the hippocampus of neonatal rats with HIBD. These results suggested that DEX treatment increased BDNF protein expression in the hippocampus was highly related to the promotion of astrocyte polarization from the A1 to the A2 state in neonatal HIBD rats.

### 3.3 DEX treatment enhanced the activation of CREB and TrkB phosphorylation and promoted hippocampal neurogenesis after HIBD

**FIGURE 3:**
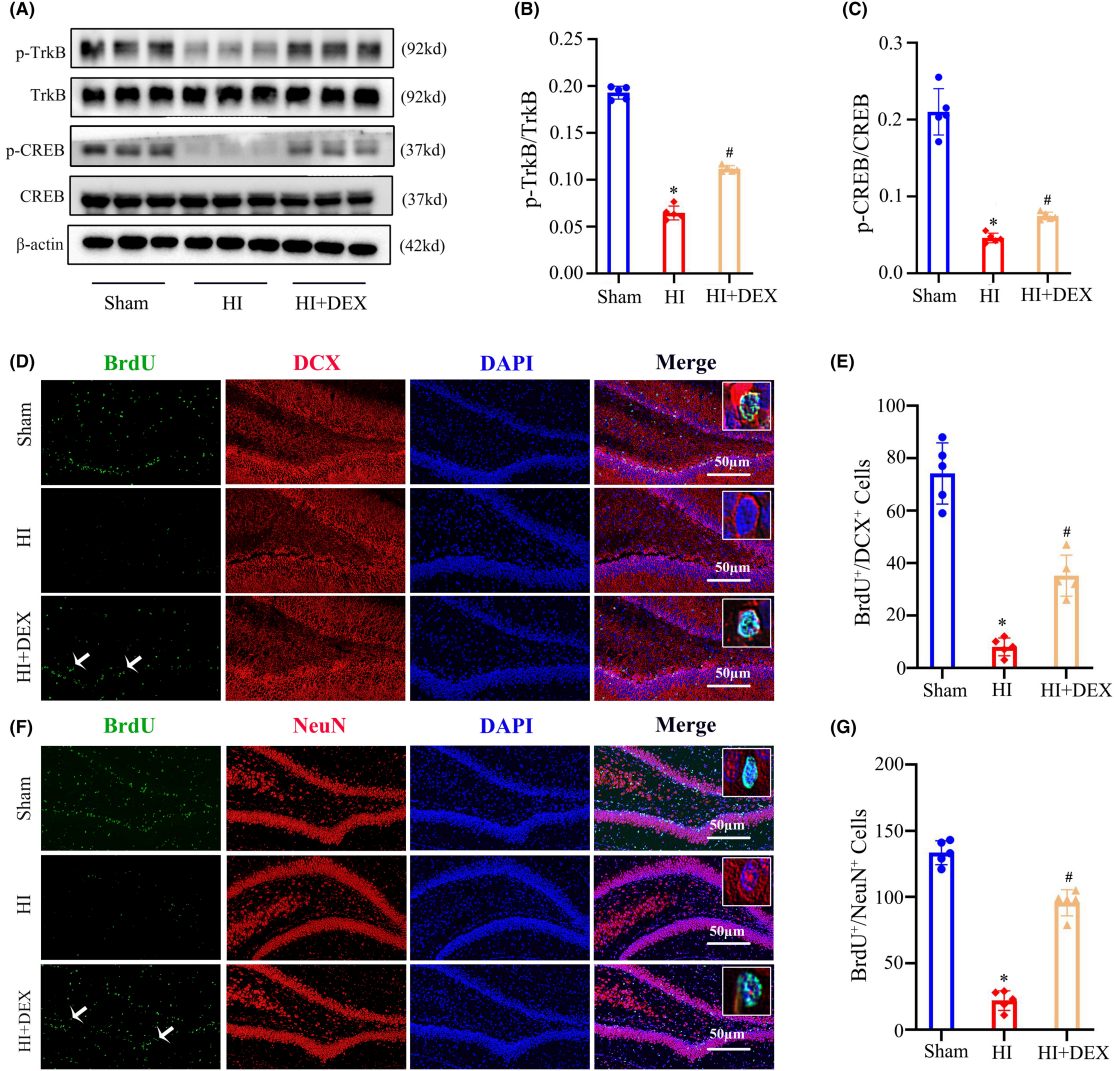
DEX treatment increased phosphorylation levels of TrkB and CREB and promoted hippocampal neurogenesis after HIBD. (A–C) Western blot analysis was performed to examine the levels of TrkB, CREB phosphorylation in the hippocampus at 2 days after HI and DEX treatment. The bar diagram represents the densitometric analyses of immunoblots. (D) Dual immunofluorescence staining was performed to examine the number of newly formed neuroblasts labeled with BrdU and DCX. (E) The quantification for the number of BrdU^+^/DCX^+^ cells in the dentate gyrus of the hippocampus. (F) Dual immunofluorescence staining was performed to examine the number of newly formed neurons labeled with BrdU and NeuN. (G) The quantification for the number of BrdU^+^/NeuN^+^ cells in the dentate gyrus of the hippocampus. DEX, dexmedetomidine; HI, hypoxic‐ischemia; HIBD, hypoxic‐–ischemic brain damage. Data were expressed as the mean ± SD (*n* = 5 per group); *p < 0.05 vs. the sham group; ^*#*^ *p* < 0.05 vs. the HI group.

As is known, BDNF/TrkB signaling triggers the activation of phosphorylated CREB, which plays a crucial role in stimulating the transcription of genes involved in hippocampal neurogenesis.^30^ To explore the potential association between DEX‐mediated neurogenesis and the activation of the BDNF/TrkB/CREB pathway in neonatal rats with HIBD, we initially analyzed the p‐TrkB/TrkB and p‐CREB/CREB ratios in the hippocampus through western blot analysis, we found that the HI group exhibited significantly reduced levels of TrkB and CREB phosphorylation compared to the sham group. However, DEX treatment demonstrated a significant ability to elevate TrkB and CREB phosphorylation levels in neonatal HIBD rats (Figure 3A–C). The levels of neurogenesis in the hippocampal dentate gyrus were then evaluated using the markers BrdU and DCX to identify newly formed neuroblasts and BrdU and NeuN to identify newly formed neurons at 14 and 28 days after HI insults, respectively. The data revealed a significant reduction in the number of newly formed neuroblasts (BrdU^+^/DCX^+^) and newly generated neurons (BrdU^+^/NeuN^+^) in the HI group compared to the sham group. However, treatment with DEX demonstrated a significant ability to enhance neurogenesis within the hippocampus of neonatal rats with HIBD, resulting in an increased number of newly formed neuroblasts and neurons (Figure 3D–G). Collectively, these results suggest a potential relationship between DEX treatment and the promotion of hippocampal neurogenesis through the activation of the BDNF/TrkB/CREB pathway in neonates with HIBD.

### 3.4 Inhibition of the BDNF/TrkB/CREB pathway reversed the ability of DEX to promote hippocampal neurogenesis after HIBD

**FIGURE 4:**
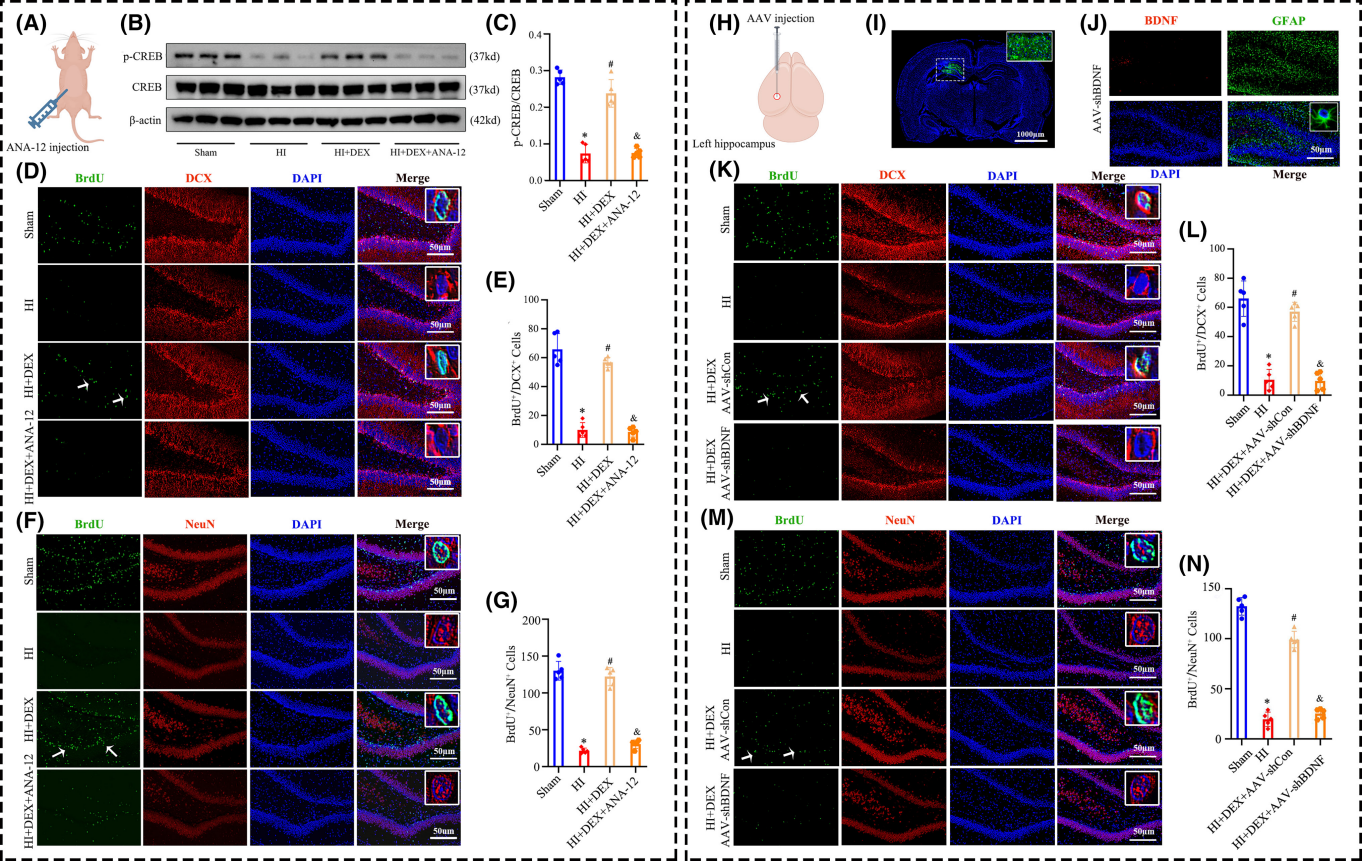
Inhibiting the BDNF/TrkB/CREB signaling pathway reversed the ability of DEX to promote hippocampal neurogenesis after HIBD. (A) The schematic illustrates the administration of ANA‐12, a selective inhibitor of TrkB, via intraperitoneal injection. (B, C) Western blot analysis was performed to examine the phosphorylation levels of CREB in the hippocampus, and the bar diagram represents the densitometric analyses of p‐CREB. (D, K) Dual immunofluorescence staining was performed to examine the number of newly formed neuroblasts labeling BrdU and DCX. (E, L) The quantification for the number of BrdU^+^/DCX^+^ cells in the dentate gyrus of the hippocampus. (F, M) Dual immunofluorescence staining was performed to examine the number of newly formed neurons labeling BrdU and NeuN. (G, N) The quantification for the number of BrdU^+^/NeuN^+^ cells in the dentate gyrus of the hippocampus. (H) The schematic illustrates the stereotactic injection of AAV‐shBDNF virus into the ipsilateral hippocampus. (I) Representative immunofluorescence image of AAV virus injection into the ipsilateral hippocampus. (J) AAV‐shBDNF virus effectively knocked down BDNF expression in the astrocytes in the dentate gyrus of the hippocampus. AAV, adeno‐associated viruses; DEX, dexmedetomidine; HI, hypoxic‐ischemia; HIBD, hypoxic‐–ischemic brain damage. Data were expressed as the mean ± SD (*n* = 5 per group); *p < 0.05 vs. the sham group; ^*#*^ *p* < 0.05 vs. the HI group; ^&^ *p* < 0.05 vs. the HI + DEX group.

To explore the involvement of the BDNF/TrkB/CREB pathway in DEX‐induced neurogenesis in neonatal rats after HIBD, the TrkB selective inhibitor ANA‐12 was administered intraperitoneally (Figure 4A) As shown in Figure 4B,C, the HI + DEX + ANA‐12 group exhibited significantly decreased levels of p‐CREB in comparison to the HI + DEX group, indicating a positive regulatory role of BDNF/TrkB signaling in CREB phosphorylation in the hippocampus following HI insults and DEX treatment. Next, we assessed the levels of hippocampal neurogenesis in the hippocampal dentate gyrus. We found a significant reduction in the number of newly formed neuroblasts (BrdU^+^/DCX^+^) (Figure 4D,E) and newly generated neurons (BrdU^+^/NeuN^+^) (Figure 4G,H) in the HI + DEX + ANA‐12 group when compared to the HI + DEX group.

Importantly, in order to specifically determine whether DEX promotes hippocampal neurogenesis by modulating the expression of BDNF in astrocytes, we selectively knocked down the BDNF gene in the astrocytes of the ipsilateral hippocampus using AAV‐shBDNF virus (Figure 4H). Using immunofluorescence staining, we observed the widespread expression of the AAV‐shBDNF virus throughout the ipsilateral hippocampus 7 days after injection (Figure 4I). Furthermore, the viral construct effectively induced knockdown of BDNF expression specifically in astrocytes within this region (Figure 4J). As expected, administering the AAV‐shBDNF virus via intrahippocampal injection successfully counteracted the positive impact of DEX on neurogenesis in the dentate gyrus following HIBD, resulting in a reduction in the number of newly formed neuroblasts (BrdU^+^/DCX^+^) (Figure 4K,L) and newly generated neurons (BrdU^+^/NeuN^+^) (Figure 4M,N). The data provided strong evidence that DEX enhances neurogenesis in the hippocampus by activating the BDNF/TrkB/CREB pathway through the polarization of A1 astrocytes toward A2 astrocytes.

### 3.5 Inhibition of the BDNF/TrkB/CREB pathway reversed the protective effects of DEX in alleviating neuronal damage and loss after HIBD

**FIGURE 5:**
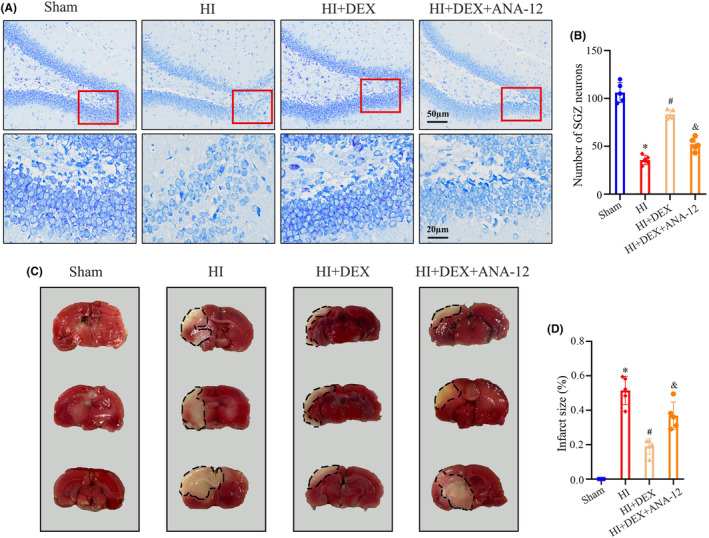
ANA‐12 treatment reversed the protective effects of DEX in alleviating neuronal damage and loss after HIBD. (A, B) The nissl staining analysis was performed to examine the number of neurons in the dentate gyrus of the hippocampus 35 days after HI. (C, D) The TTC staining analysis was performed to examine the size of cerebral infarcts 35 days after HI. DEX, dexmedetomidine; HI, hypoxic–‐ischemia; HIBD, hypoxic‐–ischemic brain damage; TTC, Triphenyl tetrazolium chloride. Data were expressed as the mean ± SD (*n* = 5 per group); *p < 0.05 vs. the sham group; ^*#*^ *p* < 0.05 vs. the HI group; ^&^ *p* < 0.05 vs. the HI + DEX group.

To further investigate the role of the BDNF/TrkB/CREB pathway in the neuroprotective effects of DEX, we conducted Nissl and TTC staining to assess neuronal damage and loss after HI insults and ANA‐12 co‐treatment. Nissl staining demonstrated significant neuropathological changes in the hippocampal dentate gyrus of the HI group compared to the sham group, including neuronal loss, nucleus shrinkage, or complete disappearance. DEX treatment effectively mitigated neuronal damage and increased the count of Nissl‐positive neurons in the dentate gyrus following HIBD. However, the protective effects of DEX in alleviating neuronal loss were reversed by ANA‐12 co‐treatment (Figure 5A,B). Consistent with the results of Nissl staining, we observed that treatment with DEX significantly reduced the sizes of cerebral infarcts following HIBD. However, this beneficial effect can be partly reversed by co‐treatment with ANA‐12 (Figure 5A,B). These results suggest that DEX‐induced neurogenesis as a critical mechanism for brain repair and regeneration involving the BDNF/TrkB/CREB pathway.

### 3.6 Inhibition of the BDNF/TrkB/CREB pathway reversed the protective effects of DEX in alleviating long‐term cognitive impairment following HIBD

**FIGURE 6:**
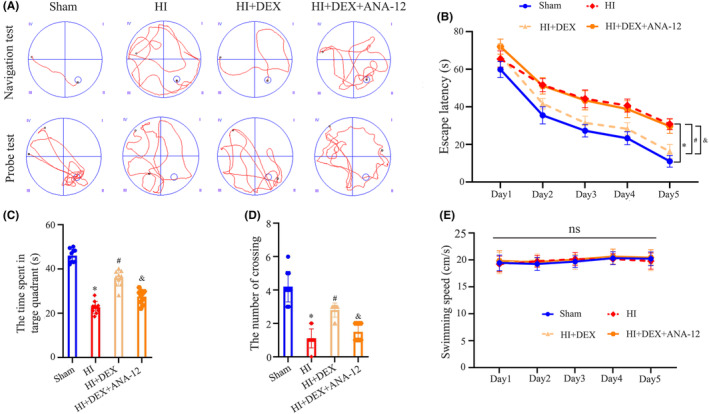
ANA‐12 treatment reversed the protective effects of DEX in alleviating long‐term cognitive impairment following HIBD. (A) Representative swimming tracks of the rats from the place navigation test on day 5 and the probe test on day 6. (B) Average escape latency to reach the hidden platform during the place navigation test over days 1–5. (C) Number of platform crossings during the probe test. (D) Time spent in the target quadrant during the probe test. (E) Swimming speeds of the rats during the probe test. DEX, dexmedetomidine; HI, hypoxic—ischemia; HIBD, hypoxic‐–ischemic brain damage. Data were expressed as the mean ± SD (*n* = 10 per group); *p < 0.05 vs. the sham group; ^*#*^ *p* < 0.05 vs. the HI group; ^&^ *p* < 0.05 vs. the HI + DEX group.

Finally, our investigation focused on determining the role of the BDNF/TrkB/CREB pathway in the long‐term cognitive impairment following HI insults and DEX treatment. In the MWM test, the swimming paths of rats on the 5th day of the place navigation and 6th day of the probe trial are depicted in Figure 1A. All groups exhibited a decrease in escape latency over time during the training session, which suggests an active learning process. In addition, our findings showed that treatment with DEX led to a decrease in escape latency time (Figure 6B), a significant increase in the percentage of time spent in the target quadrant (Figure 6C), and an increase in the number of target crossings (Figure 6D) when compared to the HI group, indicating improvement in spatial learning and memory. However, the beneficial effects of DEX were weakened when ANA‐12 was co‐treated. There was no significant difference in swim velocity among the groups (Figure 6E). These results suggested that DEX treatment remarkably alleviated HI‐induced long‐term cognitive impairment, which was partially due to the activation of the BDNF/TrkB/CREB pathway in the hippocampus.

## 4 DISCUSSION

Neonatal HIBD is still a severe and life‐threatening disease of the central nervous system that significantly contributes to neurological defects in infants and children.^1^ Multiple studies^31^, ^32^ have demonstrated altered BDNF levels following HI insults, with reported increases and decreases in various brain regions. Specifically, several of these studies^33^, ^34^ have proposed that HI insults induce neurobehavioral deficits by decreasing BDNF levels in the developing brain. Consistent with these observations, the present study confirmed a rapid decrease in BDNF levels in the ipsilateral hippocampus following neonatal HIBD. Furthermore, the study observed a significant reduction in BDNF expression primarily within astrocytes in the dentate gyrus after HI insults. The hippocampus plays a critical role in regulating cognitive function,^4^ therefore, we specifically targeted this brain region for further transcriptome sequencing analysis. The GO analysis indicated that the downregulated DEGs were mainly associated with biological processes related to nervous system development, neuron generation, and neurogenesis. Increasing evidence has demonstrated that the disruption of neurogenesis in the hippocampus significantly contributes to the development of cognitive impairment following neonatal HIBD.^35^, ^36^ The activation of the BDNF/TrkB signaling pathway plays a crucial role in reducing cell death in the vicinity of lesions, repairing neuronal damage, and facilitating neurogenesis.^37^ To identify a potential therapeutic target for treating neonatal HIBD, we investigated the neuroprotective effects of DEX on neurogenesis in the hippocampus through the BDNF/TrkB pathway.

We demonstrated that treatment with DEX effectively alleviated neuronal damage and loss in neonatal rats by promoting neurogenesis, primarily through activation of the BDNF/TrkB/CREB signaling pathway. Our findings suggested that DEX treatment ameliorated long‐term cognitive impairment caused by HI insult. This improvement was associated with an increase in the number of hippocampal neurons and a decrease in cerebral infarct volume. Furthermore, DEX treatment induced the polarization of astrocytes from the A1 to A2 phenotype and upregulated BDNF expression specifically in hippocampal astrocytes. At last, DEX treatment markedly augmented the activation of CREB and TrkB phosphorylation, leading to enhanced hippocampal neurogenesis. Inhibition of the BDNF/TrkB/CREB pathway using ANA‐12 or AAV‐shBDNF effectively reversed the pro‐neurogenic processes and neuroprotective effects induced by DEX treatment.

An appropriate animal model is crucial for investigating the underlying mechanisms and potential therapeutic targets of neonatal HIBD. In our study, we chose 7‐day‐old postnatal rats to establish the HIBD model. At this age, the rat's brain is histologically similar to that of a 32–34‐week gestational human infant or newborn.^38^ DEX's sedative and analgesic properties, minimal respiratory depression, and ability to induce cooperative sedation make it an exceptional choice for managing critically ill children in the ICU and facilitating a range of medical procedures.^39^, ^40^ Meanwhile, numerous studies^41^, ^42^, ^43^, ^44^ have demonstrated the neuroprotective effects of DEX in various brain injury conditions, including traumatic brain injury (TBI), subarachnoid hemorrhage (SAH), and cerebral ischemia. These findings highlight the potential of DEX as a promising neuroprotective agent. DEX exerts neuroprotective effects through its ability to reduce neuroinflammation, oxidative stress, apoptosis, and maintain the integrity of the blood–brain barrier (BBB).^44^ Furthermore, studies have shown that DEX can regulate cellular immunity, inhibit the infiltration of various types of immune cells into damaged nerve tissue, and modulate the reactivity of microglia and astrocytes.^45^ In this study, we demonstrated, for the first time, that treatment with DEX facilitated the conversion of astrocytes from the neurotoxic A1 phenotype to the neuroprotective A2 phenotype and enhanced BDNF expression within astrocytes in neonatal HIBD.

Astrocytes, the most abundant cell type in the brain, play crucial roles in maintaining brain homeostasis.^46^ Recent studies indicated that activated microglia can convert astrocytes into the neurotoxic A1 phenotype via the secretion of IL‐1α, TNF‐α, and C1q, and dysfunction of astrocytes critically impacts neuronal survival in various neurological diseases.^47^, ^48^, ^49^, ^50^ Previous studies, including our own, have demonstrated that DEX treatment effectively reduces microglial activation and subsequent neuroinflammation, improving neurological outcomes in neonatal rodents following HIBD.^6^, ^51^ Accordingly, we hypothesized that DEX inhibits the activation of A1 astrocytes but promotes A2 astrocyte polarization after neonatal HIBD through the inhibition of microglial activation. A2 astrocytes are widely recognized for their critical role in facilitating neurogenesis through the secretion of various neurotrophic factors, such as BDNF.^52^, ^53^ In our subsequent investigation, we employed the TrkB inhibitor ANA‐12 and AAV‐shBDNF to specifically examine the role of the BDNF/TrkB/CREB pathway in DEX‐induced neurogenesis and neuroprotection in neonatal rats after HIBD. Our findings demonstrated that inhibiting BDNF/TrkB signaling not only counteracted the positive effects of DEX in promoting CREB phosphorylation and hippocampal neurogenesis but also attenuated the neuroprotective properties of DEX in mitigating neuronal and cognitive impairments caused by neonatal HIBD.

Postnatal neurogenesis encompasses multiple stages, including NSCs proliferation, differentiation, migration to a predetermined niche, and integration into established neural circuits.^9^ Several studies^54^, ^55^ have reported a high density of proliferating cells in the subventricular zone shortly after HIBD, indicating a potential increase in subventricular neurogenesis. These exciting findings suggest that the neurogenic burst potentially acts as an adaptive response, facilitating brain recovery and enhancing neuronal replacement. However, the scope of these studies primarily focused on short‐term evaluations of cell proliferation. Due to the tightly regulated nature of the neurogenic cascade, exposure to the ischemia and other adverse environment can lead to several adverse outcomes, including increased neuroblast loss and decreased survival of newly formed mature neurons in the granular layers of the hippocampus.^56^, ^57^ In fact, it has been proposed that maladaptive neurogenesis could, in certain instances, contribute to the progression of brain dysfunction.^58^ Evaluating whether neurogenesis produces mature neurons and integrates into neural circuits to ameliorate behavioral deficits is of utmost importance. Therefore, our study aimed to investigate the long‐term process of neural stem cells (NSCs) differentiating into mature neurons in the hippocampus. Previous studies^26^ have demonstrated that cell proliferation is most active within 4–6 days after HIBD. Additionally, it is established that the process of NSCs differentiation into mature neurons typically takes 28 days. We administered BrdU injections twice daily to neonatal rats for a duration of 4–6 days following HI insults. Subsequently, we assessed the quantity of newly formed neuroblasts and neurons in the dentate gyrus of the hippocampus either 14 or 28 days post‐HI insults. Our results showed that the DEX treatment could promote the formation of mature neurons in the dentate gyrus. Collectively, our findings suggest that treatment with DEX significantly ameliorated HI‐induced cognitive impairment. These beneficial effects may, at least partially, arise from the promotion of hippocampal neurogenesis and the mitigation of neuronal damage and loss within the hippocampus.

In this study, we acknowledge several limitations. The primary limitation is the limited understanding of the specific molecular mechanisms by which DEX influences astrocyte polarization. Exploring these mechanisms will be an intriguing avenue for future research. Furthermore, cognitive function in rats was assessed exclusively using the MWM test. Additional behavioral experiments are required to comprehensively evaluate the role of BDNF signaling in HI‐induced cognitive impairment.

## 5 CONCLUSION

In conclusion, the study showed that DEX promotes hippocampal neurogenesis by activating the BDNF/TrkB/CREB signaling pathway through the induction of polarization of A1 astrocytes toward A2 astrocytes in neonatal HIBD, thereby alleviating neuronal and cognitive deficits. These findings offer an insightful perspective on the potential pharmacological mechanism involved in the neuroprotective effect of DEX.

## AUTHOR CONTRIBUTIONS

Xiaochun Zheng, Xiaohui Chen, Andi Chen, and Cansheng Gong conceived and designed the experiments., Xiaohui Chen, Andi Chen, Jianjie Wei, Yongxin Huang, Jianhui Deng, Pinzhong Chen, Yanlin Yan, and Mingxue Lin performed the experiments. Lifei Chen and Jiuyun Zhang analyzed the data. Zhibin Huang and Xiaoqian Zeng checked the manuscript for grammar and proposed some suggestions. Xiaochun Zheng, Xiaohui Chen, Andi Chen, Cansheng Gong, and Jianjie Wei wrote and edited the article.

## FUNDING INFORMATION

This research was funded by the National Natural Science Foundation of China (grant nos. 82001166 and 82171186); and the Natural Science Foundation of Fujian Province (grant nos. 2021J01366 and 2021J01385); the Training Project for Talents of Fujian Provincial Health Commission (grant nos. 2020GGA013 and 2022GGA007).

## CONFLICT OF INTEREST STATEMENT

The authors declare no competing interests.

## Supporting information


Figure S1:

## Data Availability

The datasets utilized and/or analyzed in this study can be obtained from the corresponding author upon a reasonable request.
