# Supplementary material for: Dexmedetomidine alleviates cognitive impairment by promoting hippocampal neurogenesis via BDNF/TrkB/CREB signaling pathway in hypoxic–ischemic neonatal rats
Source: CNS Neurosci Ther. 2023 Oct 13;30(1):e14486. doi: 10.1111/cns.14486 (PMC10805444; doi:10.1111/cns.14486)

Full unedited gel/blot for Figure 1C

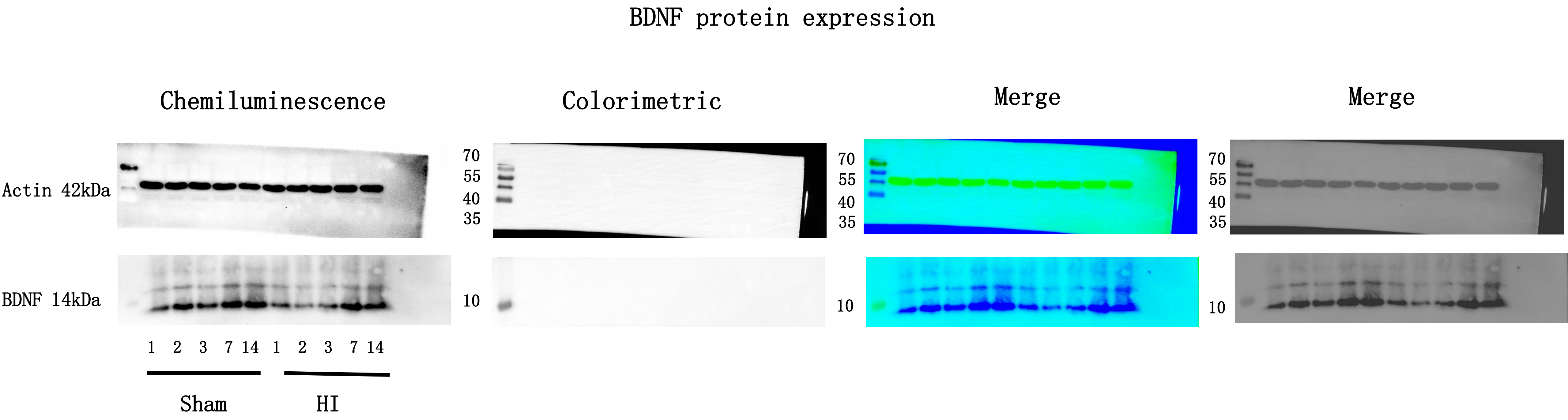

Full unedited gel/blot for Figure 1E

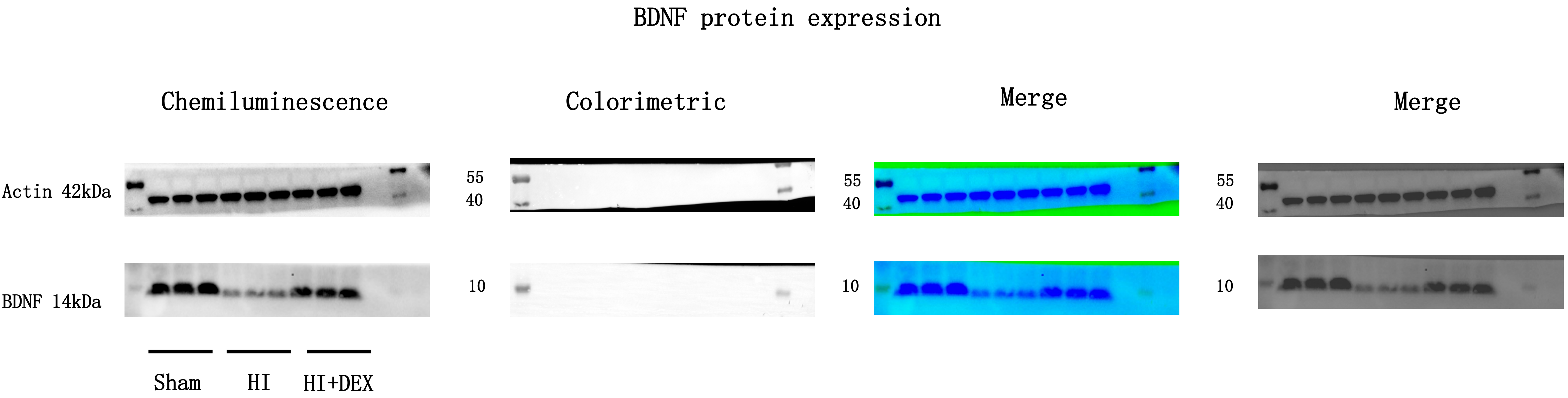

Full unedited gel/blot for Figure 3A

TrkB/P-TrkB/CREB/P-CREB

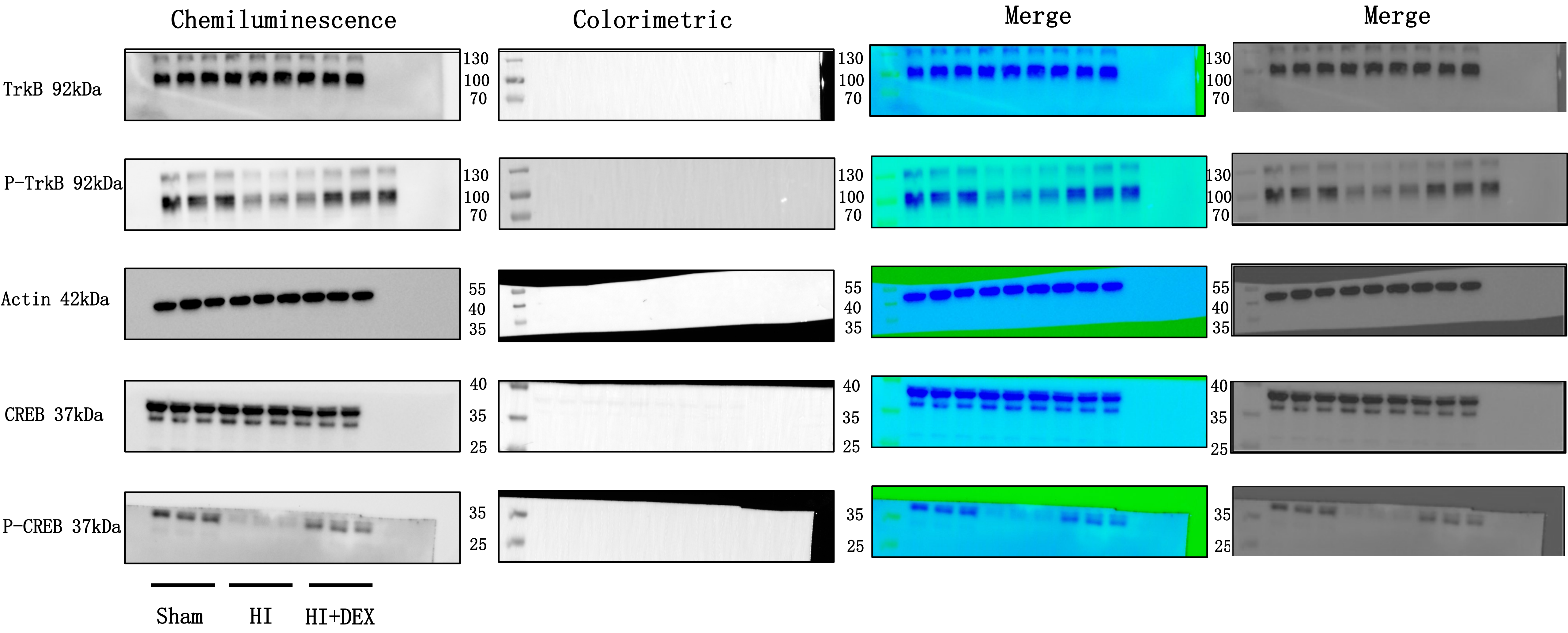

Full unedited gel/blot for Figure 4B

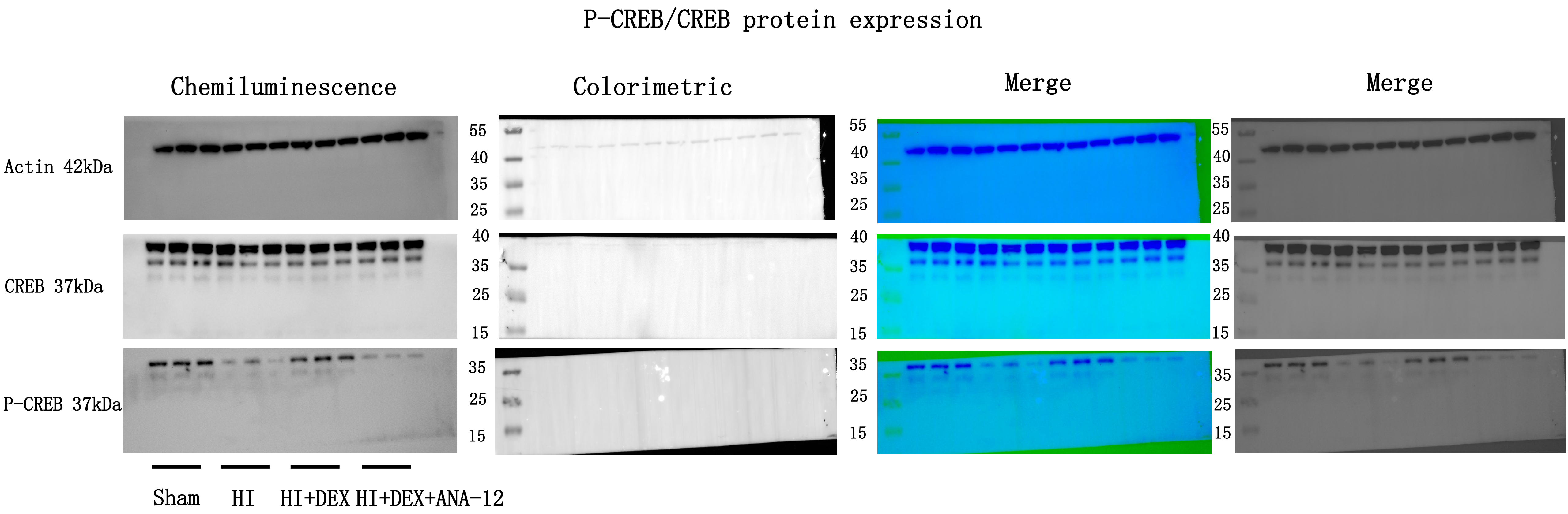

Supplement: Supplementary file 1 — Figure S1: [file CNS-30-e14486-s001.pdf]
